# Physicochemical Parameters Affecting the Distribution and Diversity of the Water Column Microbial Community in the High-Altitude Andean Lake System of La Brava and La Punta

**DOI:** 10.3390/microorganisms8081181

**Published:** 2020-08-03

**Authors:** Reynaldo Núñez Salazar, Carlos Aguirre, Jorge Soto, Pamela Salinas, Carlos Salinas, Humberto Prieto, Manuel Paneque

**Affiliations:** 1 Bionostra Chile Research Foundation, Almirante Lynch 1179, San Miguel, Santiago 8920033, Chile; rnunez@bionostra.com (R.N.S.); jsoto@bionostra.com (J.S.); psalinas@bionostra.com (P.S.); csalinas@bionostra.com (C.S.); 2 Biotechnology Laboratory, La Platina Station, Instituto de Investigaciones Agropecuarias, Santa Rosa 11610, La Pintana, Santiago 8831314, Chile; caguirre.d@gmail.com (C.A.); hprieto@inia.cl (H.P.); 3 Laboratory of Bioenergy and Environmental Biotechnology, Department of Environmental Sciences and Natural Resources, Faculty of Agricultural Sciences, University of Chile, Santa Rosa 11315, La Pintana, Santiago 8820808, Chile

**Keywords:** high altitude Andean lakes, water column, microbial diversity, physicochemical parameters, microbiome characterization, taxonomic diversity, 16S amplicon sequencing

## Abstract

Due to the low incidence of precipitation attributed to climate change, many high-altitude Andean lakes (HAALs) and lagoons distributed along the central Andes in South America may soon disappear. This includes La Brava–La Punta, a brackish lake system located south of the Salar de Atacama within a hyper-arid and halophytic biome in the Atacama Desert. Variations in the physicochemical parameters of the water column can induce changes in microbial community composition, which we aimed to determine. Sixteen sampling points across La Brava–La Punta were studied to assess the influence of water physicochemical properties on the aquatic microbial community, determined via 16S rRNA gene analysis. Parameters such as pH and the concentrations of silica, magnesium, calcium, salinity, and dissolved oxygen showed a more homogenous pattern in La Punta samples, whereas those from La Brava had greater variability; pH and total silica were significantly different between La Brava and La Punta. The predominant phyla were Proteobacteria, Bacteroidetes, Actinobacteria, and Verrucomicrobia. The genera *Psychroflexus* (36.85%), *Thiomicrospira* (12.48%), and *Pseudomonas* (7.81%) were more abundant in La Brava, while *Pseudospirillum* (20.73%) and *Roseovarius* (17.20%) were more abundant in La Punta. Among the parameters, pH was the only statistically significant factor influencing the diversity within La Brava lake. These results complement the known microbial diversity and composition in the HAALs of the Atacama Desert.

## 1. Introduction

Pristine high-altitude Andean lakes (HAALs) can be found along the central Andes of South America [1,2]. The 52 HAALs present in Chile are distributed along the Atacama Desert biome [3], including the lacustrine systems of La Brava and La Punta located in the Tilopozo sector in the extreme south of the Salar de Atacama, Antofagasta, Chile. Ecologically, La Brava and La Punta are freatogenic lakes that depend on the discharge of the Monturaqui–Negrillar–Tilopozo aquifer [4], which favors groundwater transmission and storage, with the Tilopozo sector being the only area of natural discharge [5]. In its outward trajectory, this aquifer facilitates the formation of wetlands and salty meadows and flows through a salt interface that ends up forming the shallow and saline La Brava–La Punta lake system [6]. This shallow lake ecosystem suffers from a decline in water levels resulting from permanent evaporation (137.48 ± 0.09 mm per year [5]), which causes it to recede or disappear during the dry season [7,8,9]. These dynamics result in a high salt concentration in the water column (La Brava: 62.87 ± 31.66 mS/cm; La Punta: 40.54 ± 16.57 mS/cm) [10]. In addition, other extreme conditions, such as low nutrient availability [2], influence the microbial community composition [11].

In recent years, several studies have demonstrated that several types of cultivable microorganisms exist in these extreme areas of the Atacama Desert, previously considered as a border for microbial life [12]. In particular, Atacama Desert ecosystems harbor a wide range of polyextremophilic microorganisms with broad and versatile physiological capacities. Additionally, these polyextremophiles possess peculiar biochemical pathways based on methane, sulfur, and iron, which are of great interest for industrial biotechnology [1,13,14]. Most studies identified this unique microflora through 16S rRNA gene V4 hypervariable region DNA sequencing of samples from benthic sediments of lakes in the area [14,15,16,17,18,19,20,21,22,23]. Using the 16S rRNA amplicon sequencing approach, the microflora present in extreme ecosystems can be broadly and rapidly characterized with high confidence [24]. For instance, microbial communities from several hypersaline lakes located at great altitudes around the world, such as Meyghan (Iran), Keke (China), Qaidam Basin (China), Pangong (India), Sidi Ameur and Himalatt (Algeria), and in Spain: Salobral, Salineta, La Playa, Pito, Pueyo, Guallar, Piñol, Muerte, Camarón, Rollico, Pez and Chiprana, have reported that the most predominant phyla are Proteobacteria, Bacteroidetes, Firmicutes, Actinobacteria, and in some cases Verrucomicrobia, Cyanobacteria, and Deinococcus-Thermi were present to a lesser extent [25,26,27,28,29,30]. In the Atacama Desert, this approach was used to study microbial communities from Salar del Huasco, Chungara, Cotacotani and Piacota lakes [31,32]; in that way, athalassohaline systems located in salt flats at different altitudes of the Tarapacá and Atacama regions (Chile) have shown the prevalence of Euryarchaeota, Planctomycetes, Deinococcus-Thermus, Bacteroidetes, Firmicutes, *Rhodothermaceae,* and Proteobacteria [14,15,16,17,18,19,20,21,22,23].

Meanwhile, only a few studies—based on both culture-dependent and culture-independent methods—have been carried out on the water columns of HAALs [15,19,20,23,31,32], identifying Bacteroidetes, Haloarchaea, and Proteobacteria as the main phyla. Within Proteobacteria, the most abundant classes were Alpha-, Beta-, and Gamma-Proteobacteria. However, these studies did not analyze the correlation between the composition of microorganisms and the physicochemical parameters of each lake. It is well known that physicochemical parameters such as salinity influence the composition and diversity of the microbial community in the water column [33,34,35,36]. Other factors such as dissolved oxygen (DO), pH, and nutrient concentrations also play roles in determining this diversity [12]. Thus, it is essential to understand the combined effects of physicochemical parameters associated with this type of ecosystem and their dynamics to advance our understanding of microorganism life cycles, available natural resources, and methods for preservation. Ecosystems located in the biome of the Atacama Desert, especially the La Brava–La Punta lake system which harbors diverse microorganisms, are considered to be at risk due to direct human involvement in the area [37] and climate change [5].

Elucidating the diversity and dynamics of microbial communities in response to different environmental parameters is important for uncovering novel genes, understanding the metabolism of extremophiles, and identifying protection strategies for these lake systems. Thus, the objective of this study was to determine the relationships between the physicochemical parameters of the water columns and the diversity and composition of microorganisms of this lake system. To this end, a total of 16 sampling points were established along La Brava and La Punta. Amplicon next-generation sequencing (NGS) data on the V3–V4 hypervariable region of the 16S rRNA gene collected at the end of the 2017 dry season were used to generate information about this environment during an unfavorable season. Under these conditions, we found that pH and the concentrations of silica, magnesium, calcium, salinity, and DO affect the microbial composition, but only pH and total silica were significantly different between the La Brava and La Punta sampling points.

## 2. Materials and Methods

### 2.1. Area of Study and Sample Collection

**Figure 1:**
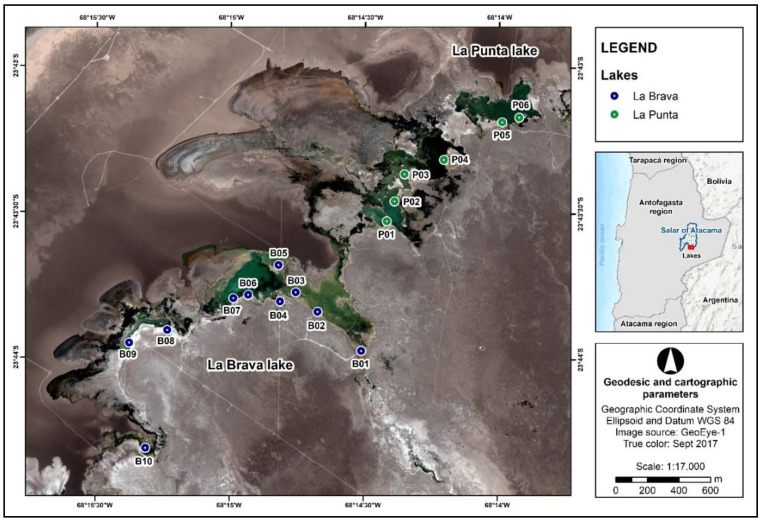
La Brava-La Punta lake system. Ten sampling points for La Brava (B points) and six for La Punta (P points) were defined according to accessibility and ecological dynamics observed in the place. Sampling points of La Brava lake are in blue (B01 to B10). Sampling points of La Punta lake in green (P01 to P06).

The study area was the high Andean lake system comprising La Brava (23°43′44″ S; 68°14′56″ W) and La Punta (23°43′29″ S; 68°14′25″ W) located in the Tilopozo sector at the southern end of the Salar de Atacama, 2305 m above the mean sea level, in the Antofagasta Region of Chile (Figure 1). The lake system is in an ecotone between wetland, salt meadow, and dry flat lake ecosystems. The latter, comprising halite (NaCl) in its nucleus, is impregnated with a brine of sodium chloride that is rich in lithium, potassium, magnesium, and boron. The marginal zone consists of a thin layer of saline sediments rich in sulfates, particularly gypsum.

The most important environmental characteristic of the La Brava–La Punta lake system is its high evaporative rate [5], which results in the reduction or disappearance of the water bodies and surrounding flooded land [6,37,38] and increases the salinity of the water column [23,38,39,40,41]. Evaporation is seasonal and increases at the beginning of summer (January–March; before the precipitation period).

The visible water inlet to the system is B01 (La Brava lake; Figure 1); from this point, the flow continues westward from La Brava lake. A bifurcation in the flow is present near B02 to the north, which permits access to La Punta lake. The water flow in La Brava lake proceeds from the southeast to the northwest, passing B02, B03, B04, B06, and B07, and followed by B08, B09, and B10 (Figure 1).

Surface water samples were collected at a depth of 5 cm at 16 representative points between December 18 and 21, 2017. The distribution of La Brava sampling points included B01 through B10, while that of La Punta included P01 through P06. These points were chosen based on accessibility and ecological dynamics, i.e., water source, which depends on conditions such as water charge and output and separates the lakes into main water bodies (La Brava: B02–B07, La Punta: P01–P04) and isolated areas (La Brava: B08–B10, La Punta: P05–P06; Figure 1; Table 1).

At each sampling point, 0.90 L of water was collected in three 0.30 L sterile polypropylene bottles. Immediately after sample collection, the bottles were closed to avoid environmental contamination [42] and placed in a refrigerated container at 4 °C for the duration of transportation to the laboratory (4 h). The samples were then stored at −20 °C for one week, during which DNA extraction was carried out.

### 2.2. Physicochemical Parameters of the Water Columns

The pH, electrical conductivity (EC), and DO of each sampling site were measured using a HI9829-1 multiparameter meter (Hanna Instruments, Woonsocket, RI, USA) [10]. Other parameters were commercially measured by Soluciones en Gestión Ambiental S.A. [*Solutions in Environmental Management*] [10] in a parallel field campaign for microbiota sampling, on the same day and at the same time and place. These parameters included total organic carbon, orthophosphate, total phosphorus, total nitrogen, nitrite, nitrate, ammonium, total suspended solids, dissolved calcium, dissolved magnesium, total silica, carbonate alkalinity, bicarbonate alkalinity, total alkalinity, and water hardness [43]. The samples were transported under optimal storage conditions with the maintenance of the cold chain until they reached the certified laboratory. Salinity values (g/L) were calculated according to the conversion method described by Williams [44], using EC values (mS/cm) with an r^2^ of 0.98. Calcium and magnesium (mg/L) were determined as dissolved ions according to the method described by Gottler [45]; while, the hardness values (CaCO_3_ mg/L) were determined by calculation, which corresponds to the sum of the total calcium and magnesium ions, as established by Baxter [46].

### 2.3. DNA Extraction

Each water sample was pre-filtered through a 25-μm filter (EMD Millipore, Burlington, MA, USA) to remove microplankton components and passed through a 3-μm pore polycarbonate membrane (Nuclepore, Kent, UK) to remove additional microplankton and nanoplankton components. Finally, the samples were filtered through a 0.2-μm-pore membrane filter (Durapore, Cork, Ireland). The filters were transferred to cryovials (Corning Life Science; Corning, NY, USA) [43] and stored at −20 °C until genomic DNA (gDNA) extraction using the BiOMICS™ DNA Microprep extraction kit (Zymo Research, Irvine, CA, USA) according to manufacturer’s instructions. Due to the low DNA concentrations, three samples per point were pooled into a composite sample. Prior to DNA sequencing, the DNA samples were quantified using a QUBIT™ 3 fluorometer (Thermo Fisher Scientific, Waltham, MA, USA). The DNA quality of the samples was verified using 1% agarose gel electrophoresis and ethidium bromide staining.

### 2.4. Sequencing and Bioinformatic Analysis

gDNA (50 ng) was fixed in DNAstable columns (Biomatrica, San Diego, CA, USA) and sent to Genewiz Inc. (South PlainField, NJ, USA) for library preparation and NGS pipeline of 16S rRNA gene sequencing using the MiSeq^®^ sequencer (Illumina, San Diego, CA, USA). gDNA libraries were constructed using the MetaVx™ kit (Genewiz Inc.). gDNA was used to generate amplicons that covered the V3 and V4 hypervariable regions of bacteria and archaea [47]. To build the libraries, adapters were added to the 16S rRNA amplicon ends using short PCR cycles. The libraries were then validated and verified using Bioanalyzer 2100 (Agilent Technology, Palo Alto, CA, USA). Sequencing was performed clockwise and counter-clockwise, with a depth of 250 base pairs (2 × 250 pair-ends). Image analysis and base readings were carried out using the proprietary software of the MiSeq^®^ sequencer.

Reads were processed and analyzed using the QIIME platform [48]. Quality control and noise elimination from reads were carried out using the ampliconnoise.py command [49]. Sequences were grouped into operational taxonomic units (OTUs) with ≥ 97% similarity using the pick_otus.py command from the Uclust method [50]. The representative sequences of each OTU were aligned using the PyNast algorithm [51] and compared with sequences in the SILVA 119 database. Subsequently, a phylogenetic tree was constructed from the aligned sequences using the FastTree method [52].

16S rRNA sequence raw data have been deposited in the NCBI nucleotide archive database under project identification number PRJNA603831.

### 2.5. Taxonomic Analysis

The number of OTUs was normalized according to a random re-sampling method as previously described [48]. OTUs were transformed into proportions and relative abundance was used to measure the composition of prokaryotic communities at the phylum, class, and genus taxonomic levels. α-diversity was calculated using different indices, including the Shannon index (entropic information on the abundance of the OTUs observed) [53], the Chao1 index (estimate of richness by species) [54], and the Simpson index (1-Dominance) [55]. Furthermore, the number of OTUs observed representing different species was determined. The Shannon index values were used to establish relationships between α-diversity and the most relevant physicochemical parameters using a Pearson correlation. For determining β-diversity, all samples were compared according to the sampling points in La Brava and La Punta lakes. Principal coordinate analysis (PCoA) was used, with the distance parameter of Weight Unifrac as a covariate [56]. In addition, the hierarchical clustering analysis WARD method was used to generate a dendrogram for the two lakes in the La Brava–La Punta system by computing the OTU values with a UniFrac distance [56].

### 2.6. Statistical Analysis

To determine the effect of the physicochemical parameters of the lake system, a Kruskal-Wallis non-parametric analysis was performed. This allowed us to establish the univariate form, the relation of the effect of each parameter in every lake and between the main and isolated bodies of each lake [57]. The taxonomic analysis was carried out using RStudio software [58] and the PhyloSeq v1.26.0 library (PhyloSeq, Stanford, CA, USA); a similarity percentages (SIMPER) analysis was performed in Vegan v2.5-6 library to explore the dissimilarities between lake factors. Summarized taxa tables at the genera levels were used to investigate the phylogenetic groups that contribute to each dissimilarity. The community distance matrix and physicochemical parameter variables were used to perform a canonical correspondence analysis (CCA) to determine the compositional variation using a permutation test under a reduced model and the constrained variables were evaluated through the chi-square test [59]. For this analysis, 31 out of 380 genera with a relative abundance >0.5% were selected and a total of 14,888 OTUs were analyzed using RStudio [57].

## 3. Results

### 3.1. Physicochemical Parameters of the Water Columns

The physicochemical parameters of the water columns in each lake are listed in Table 2. In general, La Punta displayed a more homogenous pattern of physicochemical parameter values between the sampling points, whereas greater variability was found between the tested water columns from La Brava. It appears that most of the sampling points from the main water body of La Brava (B02–B07) shared similar physicochemical parameters (Figure 1; Table 1). Some of these similarities were pH < 7.95, EC > 40.00 mS/cm, DO < 2.00 mg/L, calcium > 400.00 mg/L, and magnesium > 1300 mg/L (Table 2). These characteristics varied greatly compared with other sites that were located further away and formed an isolated body (B08–B10; Figure 1; Table 1). A similar observation was seen for La Punta when water columns from the main body (P01–P04) were compared with the isolated body (P05–P06; Figure 1; Table 1). The main differences in La Punta were found between EC, DO, and total silica (Table 2).

The salinity gradient varied widely; within La Brava, it ranged from 14.65 to 77.84 g/L while in La Punta, it was between 13.08 and 40.93 g/L. The sampling points with the highest salinity were B05 and B06, both from La Brava, each with a salinity of 77 g/L (Table 2). The pH was alkaline in both lakes and ranged from 7.62 to 8.18 in La Brava and from 8.07 to 8.35 in La Punta. The most alkaline site was P03 (pH = 8.35) from La Punta, whereas the lowest pH was found in B05 (pH = 7.62) from La Brava. The DO levels displayed a wider range within La Brava (1.58–6.35 mg/L) than in La Punta (3.14–4.25 mg/L). Interestingly, all hypoxic sites (DO < 2.00 mg/L; B02, B03, B04, and B06) were located in La Brava (Table 2).

Dissolved ions calcium and magnesium levels ranged from 219.88 to 835.40 mg/L and 549.00 to 4545.00 mg/L, respectively, in La Brava and from 242.26 to 484.50 mg/L and 397.08 to 1141.20 mg/L, respectively, in La Punta. On the other hand, silica concentrations varied greatly between both lakes; within La Brava, silica levels ranged from 68.90 up to 91.80 mg/L, whereas these concentrations started at 100.50 and reached 127.60 mg/L in La Punta (Table 2). Regarding carbonate alkalinity, no major variation was observed in La Brava, except for the sampling points B08 and B09 (isolated bodies) that exhibited high concentrations (46.90 and 89.50 mg/L, respectively) similar to those observed for the main body of water in La Punta (P01–P04; Table 1; Table 2). The total organic carbon levels were similar between both lakes, with 5.00–17.40 mg/L and 5.84–15.40 mg/L in La Brava and La Punta, respectively (Table 2).

According to Kruskal-Wallis analysis, the only physicochemical parameters that showed a significant difference between La Brava and La Punta lakes were pH (*p* = 0.0009) and total silica (*p* = 0.0002; Table S1). However, when comparing the main bodies of both lakes, significant differences were observed between them in terms of pH (*p* = 0.0061), DO (*p* = 0.0121), salinity (*p* = 0.0242), EC (*p* = 0.0242), total silica (*p* = 0.0061), dissolved magnesium (*p* = 0.0242), hardness (*p* = 0.0242), and carbonate alkalinity (*p* = 0.0030). In contrast, no significant differences were observed for any parameter between the isolated bodies of both lakes (Table S1). Furthermore, a significant difference was observed in DO (*p* = 0.0167) between the main and isolated bodies of La Brava, while no significant differences were observed between those of La Punta (Table S1).

### 3.2. Next-Generation Sequencing Data

Data from DNA extraction and NGS sequencing of the samples was used to generate a database consisting of 27,582,598 raw reads from the two lakes. These reads were 250 bp long and contigs covered the V3–V4 hypervariable region of the 16S rRNA gene. The average distribution of this data was 1,831,622 ± 221,609 raw reads for La Brava and 1,544,397 ± 378,115 for La Punta. The average distribution without chimeras was 724,678 ± 91,478 for La Brava and 584,398 ± 166,009 for La Punta. Quality refined sequence libraries were clustered into 14,888 OTUs across all samples (Table S2).

### 3.3. Diversity Comparisons

To determine α-diversity, the sequences were grouped into OTUs with a similarity of ≥ 97.00% to calculate diversity and richness indices. Sites with the highest Chao index richness values were B01 (1234) and B10 (1235) for La Brava and P02 (1071) for La Punta. However, those with the highest α-diversity obtained via the Shannon index were B01, B09, P01, and P03 (4.24, 4.20, 4.25, and 4.22, respectively), while those with the lowest were B04, B10, and P05 (3.67, 3.43, and 3.98, respectively; Table 3; Figure S1).

**Figure 2:**
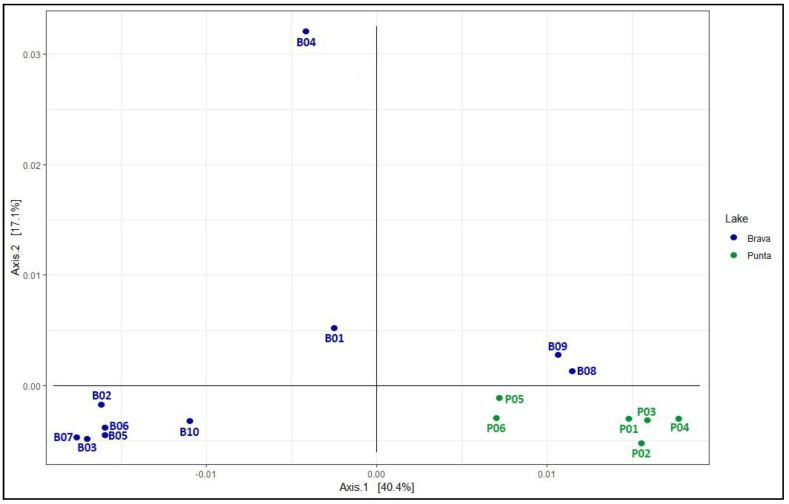
Principal coordinate analysis by the Bray Curtis distance of dissimilarity. The Unifrac distance of weight was considered as a covariate.

**Figure 3:**
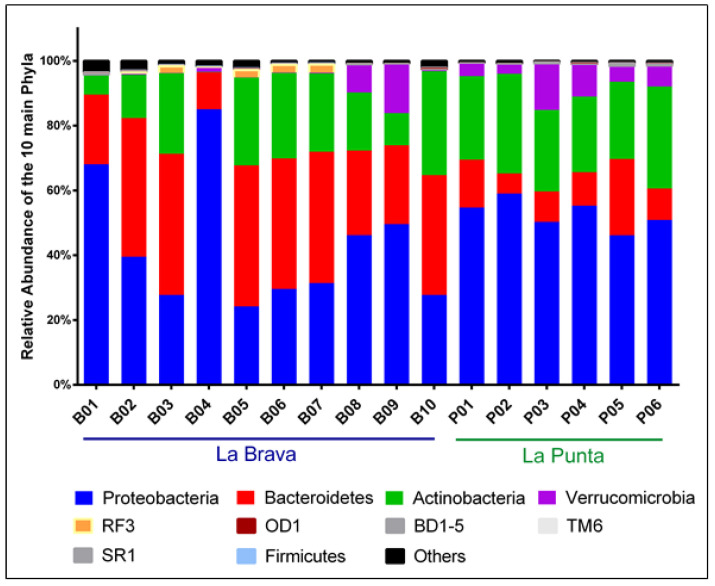
Microbial community composition of the sampling points from La Brava and La Punta lakes. The relative abundance of the main phyla across the 16 water column samples collected from the La Brava–La Punta lake system based on massive sequencing data of the V3–V4 hypervariable region of the 16S rRNA gene. Ten phyla are responsible for 96.41% to 99.63% of the relative abundance observed.

β-diversity analysis revealed two separate groups at a distance of 1.40 (Figure S2). The first group included all sampling points from La Punta as well as B08 and B09 from La Brava. The second group consisted of the remaining points from La Brava lake. Within the first group, there were two clusters of P01–P04 and B08, B09, P05, and P06. The remaining points from La Brava lake formed two subgroups: the first included B02, B03, B05, B06, and B07 and the other included B01, B04, and B10. PCoA analysis (Figure 2) revealed a total variance of 57.50%, considering the two axes. The first axis ratified the groupings obtained in the dendrogram (Figure 2; Figure S2). The β-diversity on the first axis (40.40%) divided the points from La Brava lake into three groups: B08 and B09; B01, B04, and B10; and B02, B03, B05, B06, and B07. For La Punta lake, two groupings of P05–P06 and P01–P04 were evident. On the second axis (17.10%), B01, B04, B08, and B09 were distinct from the remaining La Brava sampling points (Figure 3).

Correlations between the Shannon index and the physicochemical parameters of the water columns in the La Brava–La Punta lake system (as a whole) were explored, and a significant relationship between pH, EC, salinity, and carbonate alkalinity was observed (Table S3). However, when analyzing each lake separately, only a significant relationship was observed between the Shannon index and pH in La Brava (r = 0.75, *p* = 0.0116; Table S3). When evaluating the physicochemical parameters of the water columns according to the main and isolated body groupings of both lakes (Table S3), no significant correlations were observed.

The microbial communities in both lakes were predominantly composed of Proteobacteria, Bacteroidetes, Actinobacteria, and Verrucomicrobia, which comprised more than 96.00% of all OTUs (Figure 3). The RF3 phylum accounted for 0.60% of total relative abundance. The phyla Proteobacteria, Bacteroidetes, Actinobacteria, and Verrucomicrobia accounted for 42.93 ± 19.99%, 33.11 ± 11.70%, 18.18 ± 10.51%, and 2.56 ± 5.06% in La Brava, respectively; and 52.75 ± 4.54%, 26.73 ± 3.51%, 12.33 ± 6.14%, and 6.84 ± 4.29% in La Punta, respectively (Figure 3). More specifically, Flavobacteria were on average ~3.59 times more abundant in La Brava lake while Verrucomicrobia and Cytophagia were 2.70 and 4.56 times more abundant in La Punta (Figure 3).

### 3.4. Comparison between Lakes at the Genus Level

An analysis of the most representative OTUs defining the lake system was performed using SIMPER (Table 4) with the 100 most abundant OTUs in each lake. The results showed that dissimilarities between the lakes could be attributed to 11 OTUs. These genera contributed 75.36% of the dissimilarities between both lakes and included *Psychroflexus*, *Thiomicrospira*, *Pseudospirillum*, *Pseudomonas*, *Roseovarius*, *Ruegeria*, *Pseudoalteromonas*, *Rubritalea*, *Candidatus_Aquiluna*, *Roseibaca*, and *DS001*. The dissimilarities were largely driven by *Psychroflexus* (26.37%); this genus is part of the Flavobacteria class of phylum Bacteroidetes. The other genera belong to Gamma-proteobacteria (*Thiomicrospira*, *Pseudospirillum*, *Pseudomonas*, and *Pseudoalteromonas*), Alpha-proteobacteria (*Roseovarius*, *Ruegeria*, and *Roseibaca*), Actinobacteria (*Candidatus_Aquiluna* and *DS001*), and Verrucomicrobia (*Rubritalea*). Unlike previous results, the relative abundance of the class Cytophagia in all La Brava sampling points was < 2.00% but it ranged from 2.44% to 5.19% in La Punta.

SIMPER analysis was also used to highlight differences between the different areas of the lakes. When comparing the main and isolated water bodies of La Brava lake, 13 genera could explain 75.09% of the differences between these sections; 52.08% of the dissimilarities were attributed to *Psychroflexus*, *Pseudospirillum*, *Rubritalea*, *Pseudomonas*, and *Pseudoalteromonas* (Table 4). In La Punta lake, only 9 genera were responsible for 75.13% of the differences between the main and isolated water bodies. Within La Punta, the main contributing genus was not *Psychroflexus* but *Thiomicrospira*, which was responsible for 20.51% of the dissimilarities. Moreover, 56.69% of the differences were attributed to the genera *Thiomicrospira*, *Psychroflexus*, and *Pseudomonas* (Table 4).

When comparing the main bodies of La Brava and La Punta, a total of 10 genera contributed to 75.05% of the dissimilarities between them; these differences exhibited a similar pattern to that of the whole lakes when compared, except for the absence of *Candidatus_Aquiluna* (Table 4). The comparison between the isolated bodies of La Brava and La Punta indicated that 11 genera contributed to 76.01% of the dissimilarities (Table 4). In contrast to the previous analyses, the *NS5 marine group* was present instead of the genus *DS001* and 53.63% of the dissimilarities were attributed to *Psychroflexus*, *Pseudomonas*, *Pseudospirillum*, and *Rubritalea*.

**Figure 4:**
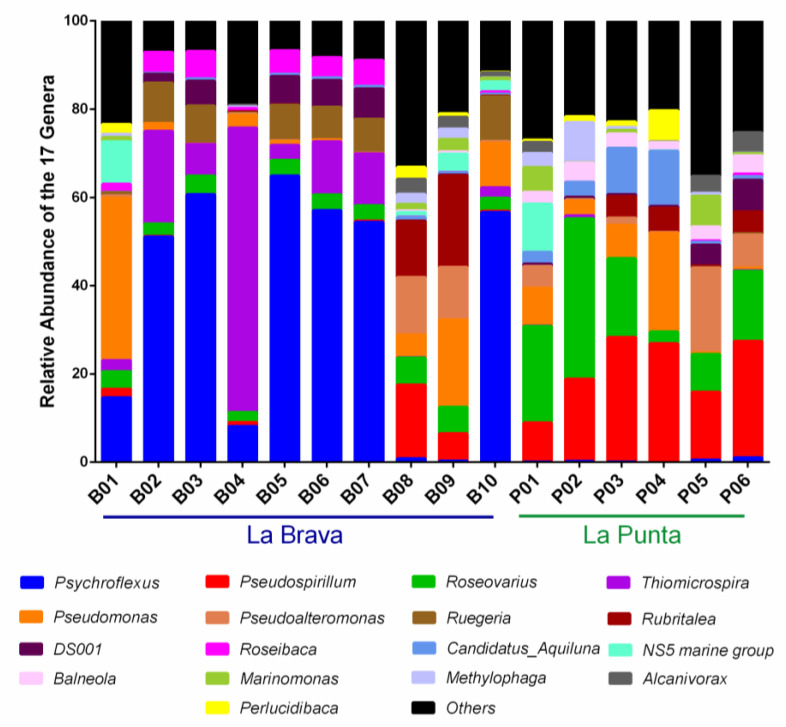
Relative abundance of the genera in the 16 water column samples collected from the La Brava–La Punta lake system. Seventeen genera are responsible for 64.80% to 93.31% of the relative abundance observed.

With the exception of B08 (0.83%) and B09 (0.31%), La Brava lake generally had a higher relative abundance of the genus *Psychroflexus* (36.85 ± 27.11%) than that of La Punta lake (0.32 ± 0.39%). At B08 and B09, the genera *Ruegeria*, *DS001*, and *Roseibaca* had an average relative abundance of 0.01 ± 0.00%, 0.11 ± 0.01%, and 0.04 ± 0.04%, respectively. These three genera had a significantly lower abundance at these sites in comparison with the rest of La Brava lake, with an average abundance ranging from 3.39 ± 3.15% to 6.49 ± 3.82% (Figure 4; Table S4).

On the other hand, the genera *Pseudoalteromonas*, *Rubritalea*, *Marinomonas*, *Methylophaga*, and *Alcanivorax* were predominant at B08 and B09. Differences in the microbial community on the genus level were also seen at B01 (inlet). Compared with the other sample locations from La Brava lake, the genus *Pseudomonas* (36.81%) was predominant and the *NS5 marine group* was prevalent (9.63%) at B01. The genus *Thiomicrospira* was predominant only at B04 (64.45%) compared with the other sampling points (0.01% to 20.94%). The genera *Pseudospirillum*, *Roseovarius*, and *Balneola* had an average relative abundance of 20.73% ± 7.75, 17.20% ± 11.64, and 3.24% ± 0.91, respectively, in La Punta (Figure 3; Table S3). Other abundant genera such as the *NS5 marine group*, *Candidatus_Aquiluna*, *Marinomonas*, *Methylophage*, and *Alcanivorax* exhibited a site-specific pattern throughout La Punta (Figure 4; Table S4).

A difference was observed for the average relative abundance of the genus *Pseudoalteromonas* between lakes, which was almost twice as high in La Punta (5.62 ± 7.23%) compared to La Brava (2.64 ± 5.18%), but with a high standard deviation. However, the relative abundance of the genus *Pseudoalteromonas* at the two isolated sample locations B08 and B09 was 13.03% and 11.97%, respectively (Figure 4; Table S4).

In La Punta, the genus *Pseudospirillum* had a higher average relative abundance (20.73 ± 7.75%) than at La Brava (2.66 ± 5.28%). The minimum relative abundance was observed in sampling point P01 (8.83%) and the maximum at P03 (28.23%), while the minimum and maximum relative abundance were detected at sampling points B05 (0.02%) and B08 (16.70%), respectively, in La Brava. Similarly, the genus *Roseovarius* had a higher relative abundance average (17.20 ± 11.64%) in La Punta compared with La Brava (3.88 ± 1.28%; Figure 4; Table S4).

The genus *Candidatus_Aquiluna* was identified in all sampling points of La Punta lake, with a relative abundance between 0.71 and 12.54%; meanwhile, the relative abundance did not surpass 1.00% at La Brava. The genus *Balneola* was only identified in five La Brava sampling points (B01, 0.01%; B04, 0.01%; B08, 0.40%; B09, 0.52%; and B10, 0.01%), with < 1.00% relative abundance (Figure 4; Table S4).

Overall, sampling locations B08 and B09 of La Brava showed a genus-level distribution pattern similar to that of the La Punta water column (Figure 2 and Figure 3).

### 3.5. Distribution of Genera with Respect to Environmental Variables

**Figure 5:**
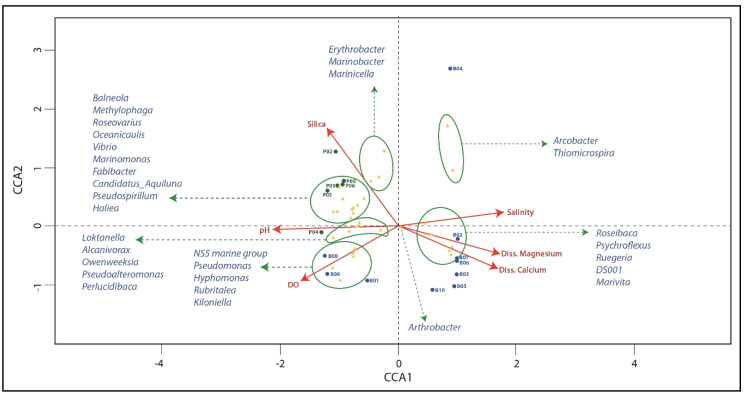
Canonical correspondence analysis (CCA) of the distribution of the most abundant genera with respect to six environmental variables. Samples are indicated with B for La Brava (blue dot) or P for La Punta (green dot). Genera are represented by yellow triangles and environmental variables by red arrows (average relative abundance of the genera > 0.5%).

Next, we performed CCA that included all physicochemical parameters (Table 2). Environmental parameters that were found statistically insignificant by the chi-square significance test were excluded from further analyses. As a result, only six physicochemical variables were predominant: silica, pH, DO, salinity, and dissolved calcium and magnesium. The total inertia was 2.06 while the constrained inertia was 1.25, indicating that the constrained variables can explain 60.00% of the variation in the distribution of known genera. Finally, the first two components of constrained eigenvalues exhibited proportions of 57.20% and 22.80%, respectively. The sampling points within the first component of the CCA were divided into two groups: the first consisted of B02–B07 and B10, which were associated with salinity, magnesium, and calcium, while the second group consisted of P01–P06 as well as B01, B08, and B09 and were associated with silica, pH, and DO (Figure 2 and Figure 5).

The genera Erythrobacter, Candidatus_Aquiluna, Rubritalea, NS5 marine group, Owenweeksia, Fabibacter, Balneola, Hyphomonas, Pseudospirillum, Perlucidibaca, Haliea, Alcanivois, Marinicella, Pseudoalteromonas, Vibrio, Marinobacter, Loktanella, and Roseovarius were positively associated with silica, pH, and DO, as their relative abundance increased with an increase in these parameters. Meanwhile, the genera Ruegeria, Marivita, DS001, Arthrobacter, Psychroflexus, Arcobacter, Roseibaca, and Thiomicrospira were positively associated with salinity, magnesium, and calcium (Figure 5).

Overall, the groups formed by sample location based on physicochemical parameters (Figure 5) were highly similar to those formed by β-diversity analysis (Figure S2). The genus *Marivita* and the sampling points B02–B07 and B10 from La Brava were all moderately influenced by salinity. In addition to salinity, B02, B06, and B07, which were closely associated with the genera *Roseibaca*, *Psychroflexus,* and *Ruegeria*, were best described according to calcium and magnesium levels. Sampling points B01, B08, and B09 were best described according to DO, similar to the genera *NS5 marine group*, *Pseudomonas*, *Hyphomonas*, *Rubritalea,* and *Kiloniella*. Meanwhile, the genera *Arcobacter* and *Thiomicrospira* as well as hypoxic sampling points (Table 2), specifically B04, were negatively associated with DO. Silica best-described P02 and moderately influenced P01, P03, P05, and P06. pH best-described P04 and the genera *Loktanella*, *Alcanivorax*, and *Pseudoalteromonas*. The genera *Marinobacter* and *Marinicella* were best explained by high silica concentrations. Lastly, ten genera, which were mostly associated with Proteobacteria (n = 7) and clustered more closely in sample locations in lake La Punta, were influenced by both pH and salinity (Figure 5).

## 4. Discussion

Several studies have explored the diversity of microorganisms in the water and sediments along the Salar de Atacama and found that the most prevalent phyla were Proteobacteria, Cyanobacteria, and Bacteroidetes [17,21,22,23,59,60,61]. The present study focused on characterizing the microbial composition of the La Brava–La Punta lake system’s water, with an increased effort in the sampling of La Brava lake and including the previously uncharacterized La Punta lake. The study area exhibits complex environmental dynamics; however, the evapotranspiration values remain stable under the annual dry season conditions. In December of 2010–2017, the area had an average evapotranspiration rate of 189.47 ± 5.11 mm/month; specifically, the values for December 2016 and 2017 were 189.65 and 183.08 mm/month, respectively [5]. It should be noted that the La Brava–La Punta lake system is fed by a constant flow of water from an aquifer, which can result in biogeochemical conditions that adapt to a dynamic ecosystem [6,22,39]. As a consequence, the diversity and composition of microbes varied at different sampling points depending on the physicochemical parameters that result from the recharge and discharge of the lake system and the isolated bodies formed during the dry season [7,62].

### 4.1. Physicochemical Parameters of Water and Microbial Diversity

The environmental parameters varied depending on which sections of the lakes were considered for the analysis. Initial comparisons between both lakes (La Brava B01–B10 and La Punta P01–P06) revealed that only pH and total silica were significantly different (Table S1). But when each lake was broken down into different sections (main body and isolated water bodies) other parameters also varied significantly (Table S1). Most of these significant differences in physicochemical parameters (pH, DO, EC, salinity, dissolved magnesium, silica, carbonate alkalinity, and hardness; Table S1) were detected between the main body and La Brava and also the main body and La Punta.

Regarding the pH, it was possible to differentiate between sampling points with pH ≥ 8.00 or pH < 8.00. In La Brava, most of the main body (B02–B07) had a pH < 8.00, whereas all sampling points in La Punta had a pH ≥ 8.00. Interestingly, these same zones were also defined based on values for other physicochemical parameters of the water columns, such as EC, DO, dissolved calcium, dissolved magnesium, and carbonate alkalinity (Table 2).

Total silica concentrations in La Brava showed significant differences between the main and isolated bodies (*p* = 0.8333, Table S1), but no significant differences were observed in La Punta. Previous studies have indicated that total silica can explain the significant variations in the structures of bacterial communities in shallow hydrothermal water sediments, favoring the *Pseudomonas* and *Pseudoalteromonas* genera [63].

In terms of α-diversity, the highest values were observed in La Punta lake, particularly in the main body (P01–P03). In La Brava, the water entry point (B01) and the isolated bodies B08 and B09 exhibited the highest values (Table 3; Figure S1). Our results are in contrast with previous studies on the water columns of four different Chilean HAAL systems, which reported Shannon indexes lower than 2.90 [16]. Our values also differed from another study on the Salar del Huasco (α diversity varies between 0.70 and 1.70) [32], a salt flat located north of the La Punta-La Brava lake system that is dotted with ponds and salt marshes and is seasonally partially covered with water. The differences can be explained by the experimental approaches used as denaturing gel electrophoresis (DGGE) and enrichment culture medium are less sensitive in terms of amplification and subsequent visualization of the microbial community. Indeed, our findings are consistent with other studies on the Salar del Huasco that also employed massive sequencing approaches [32].

When comparing the diversity between both lakes, the β-diversity analysis revealed a clustering of B02, B03, B05, B06, and B07, forming a group that corresponds to the main body of La Brava (Figure 1; Table 1; Figure 4). B08 and B09 (La Brava) showed similar features to those of P05 and P06 (La Punta; Figure 4), with similar alkaline pH values that differed from those recorded in the main bodies of both lakes (Table 2). In La Punta, the β-diversity analysis also identified one main body comprised of P01–P04 (Figure 4), which displayed similar EC, total nitrogen, and carbonate levels (Table 2) and differed from the subgroup formed by P05 and P06 (Table 1; Figure 4).

### 4.2. Distribution of Genera with Respect to Environmental Variables

Our water column analysis results are in agreement with previous observations and highlighted the prevalence of the phyla Proteobacteria and Bacteroidetes, which represented an average relative abundance > 96.00% in the system. Furthermore, Actinobacteria and Verrucomicrobia were found to be more abundant in La Punta than in La Brava. In contrast, sediment characterizations of Tebenchique [21] and La Brava [23] reported Firmicutes as a major phylum, while Proteobacteria, Bacteroidetes, and Actinobacteria were less abundant. Noticeable differences between water and sediment microbial communities also included a much lower abundance of Planctomycetes, Chloroflexi, Deinococcus Thermus, and Spirochaetes in the water column than in the sediment (Figure 3) [21,23].

When we compared our results at the taxonomic phylum level with those of other hypersaline lakes from around the globe, we noticed that the microbial composition of the La Brava-La Punta lake system was most similar to the composition of the lakes in Iran [25], India [28], China [64], and Spain [30], all of which show a predominance of Proteobacteria followed by Bacteroidetes, Actinobacteria, and Firmicutes. In the case of the saline shallow lakes of the Monegros Desert in Spain [30], *Psychroflexus* was the most abundant genus, similar to that of La Brava–La Punta (Figure 4; Table S4).

As physicochemical patterns define the microbial community structure in particular regions of these lake systems, we further assessed the differences between the main microbial communities as well as their specific environmental drivers. SIMPER analysis showed that *Psychroflexus*, *Thiomicrospira*, *Pseudospirillum*, and *Pseudomonas* accounted for up to 52.77% of the differences, while *Psychroflexus* by itself accounted for 26.37% (Table 4). *Psychroflexus*, *Thiomicrospira*, *Pseudospirillum*, and *Pseudomonas* are aerobic and chemoheterotrophic bacteria [65,66,67], and with exception of *Pseudomonas,* they require high salinity levels for proper growth [65,66,67]. Similar results were obtained via SIMPER analysis when comparing different sections of the lakes (Table 4) as *Psychroflexus* accounted for the most differences (18.48% to 23.87%); the only exception was in the case of the La Punta main body versus the isolated water bodies, as 20.51% of the dissimilarities were attributed to *Thiomicrospira*. Moreover, higher participation of the genus *Rubritalea* was identified when comparing the main body with the isolated bodies of La Brava (37.55%), and the isolated bodies of La Brava and La Punta (53.63; Table 4).

High salinity levels were characteristic of both lakes; however, pH was the differentiating environmental factor. This was clearly reflected in the abundance of the genera *Psychroflexus* and *Thiomicrospira*. Both, particularly *Psychroflexus*, favor a pH range of 7.00–7.50 for optimal growth [65,66]. Indeed, these low pH ranges and genera were found exclusively in La Brava at B02–B07 and B10 (Table 2; Figure 4), with the highest relative abundance of *Psychroflexus* (64.90%) identified at B05. The genus *Psychroflexus* has been isolated from water columns of the hypersaline (90.00 g/L) Xiaochaidan Lake located in the Qiadan basin (China), and it is described to grow optimally at neutral pH [68]. Thus, these results support the notion that pH and salinity facilitate the prevalence of certain microbial genera.

Compared with previous works characterizing sediment samples from La Brava lake [22,23], there appears to be a few similarities in the bacterial compositions of the microbial communities, such as the significant abundance of the phyla Proteobacteria, Bacteroidetes, and Verrucomicrobia (Figure 3). Despite these similarities, there were some noticeable differences, including (1) the presence of the phylum Actinobacteria in both lakes, which was not observed in the sediment samples, and (2) the greater presence of Planctomycetes, Chloroflexi, Deinococcus-Thermus, and Spirochaetes in the sediment. These differences in predominant phyla highlight the relevance of the type of sample used when analyzing the environmental microbial composition.

The influence of silica on the genera *Marinobacter*, *Marinicella*, *Methylophafa*, *Marinimonas*, and *Haliea* (Figure 5) can be associated with the metabolic processes that allow hard silica to precipitate in lake sediments, as suggested by Sauro et al. [69]. In their work, water bodies containing silica were analyzed, showing a predominance of chemotrophic microbial communities of the phylum Proteobacteria and different classes, among which the class Gamma-proteobacteria was predominant. These communities were associated with the biomineralization processes of silica, which causes its precipitation from an aqueous amorphous state to a hard-solid state. This process occurs through an increase in pH mediated by microbial metabolism, e.g., nitrogen fixation, decomposition of proteins or amino acids, degradation of urea, and consumption of CO_2_ [69]. Silica can also be solubilized by different species of the genus *Pseudomona* [70], which happens to be more abundant in the main body of La Punta (P01-P04) and in the isolated bodies of La Brava (B08-B10).

Meanwhile, the genus *Marivita* is associated with high-salinity sites. This genus has been identified in the seawater of the coasts of the Republic of Korea [71], temperate estuaries [72], and salt lakes in China [73]. The presence of this genus in the La Brava–La-Punta lake system likely results from the meso and/or hypersaline conditions observed for all points sampled in the present work (Table 2; Figure 5).

The La Brava–La Punta lake system can be classified as a predominantly oxy-type environment, as DO levels were above 2.00 mg/L for most of the sampling points analyzed. This condition favors the prevalence of aerobic-type bacterial microorganisms, such as *Rubritalea*, *Kiloniella*, *Pseudonomas*, *Hypnomonas*, and *Thiomicrospira* [66,67,74,75,76]. In hypoxic sites, however, there was a predominance of the genera *Thiomicrospira* and *Arcobacter* (Table 2, Figure 5). *Arcobacter* possesses a microaerophilic-type metabolism, meaning that optimal growth occurs in environments with low DO concentrations [77]. Despite being an aerobe, *Thiomicrospira* can also grow in environments with low oxygen saturation [66]; thus, it makes sense that both genera were found in the hypoxic zones of the lake system (all within La Brava: B05, B07, B08, and B09). The presence of these two genera in the hypoxic zones can also be explained by their importance in the sulfur cycle as they both act as sulfur-oxidizing agents in aquatic environments [78,79]. The sulfur cycle and the oxidation of sulfide (S^2-^) and hydrogen sulfide (H_2_S) are carried out in transition environments between anoxic (devoid of DO) and oxic; therefore, hypoxic sites are the ideal zones for the growth of bacteria associated with these metabolic processes [79].

Previous studies on the composition and changes in the aquatic microbial diversity of terrestrial aquifers [79] and of hypersaline lakes [80] have established that both calcium and magnesium exert critical roles on the microbial community composition. The genera *Roseibaca*, *Psychroflexus*, *Ruegeria*, and *DS001* have also been detected and isolated by other groups from marine waters [81,82,83] and hypersaline lakes [65], which are environments known to have high concentrations of sodium, chlorine, calcium, and magnesium—characteristics similar to those of La Brava and La Punta. The concentrations of Ca^2+^ and Mg^2+^ ions present in the water column can be affected by processes of biomineralization and bioprecipitation resulting from the presence of certain bacteria in these environments, which lower the ion concentration in the water [84,85,86]. Biomineralization requires relatively high pH levels [85,86]. Moreover, it has been well documented that the aerobic bacteria of genus *Bacillus* and *Pseudomonas* are responsible for the bioprecipitation of Ca^2+^, Mg^2+^, and carbonates in different sources such as seawater, hypersaline lakes, sediment, and soil [84,85,86,87,88]. In the La Brava-La Punta lake system, the genus *Pseudomonas* was detected in B01 (water inlet to La Brava) and B08–B10 (isolated bodies) as well in the main body of La Punta (P01–P04; Figure 4; Table S3). The presence of *Pseudomonas* also coincided with the sections of the lake system that had relatively higher pH levels (pH > 8.00) and DO concentrations (3.09–6.35 mg/L) as well as the lowest concentrations of dissolved calcium (219.88–358.44 mg/L) and magnesium (397.08–1070.50 mg/L; Table 2). These parameters facilitate the successful bioprecipitation of Ca^2+^ and Mg^2+^ ions present in the lakes by *Pseudomonas* and can very likely explain the lower concentration of these ions in these sampling points.

Furthermore, the presence of the genera *Pseudomonas*, *Legionella*, *Rhodopseudomonas*, and *Luteimonas* in our sampling points support the roles of decomposition or digestion of organic matter [89], while *Thiomicrospira*, *Bacillus*, *Acinetobacter*, and *Halomonas* allow for carbon precipitation [90]. The genera *Thiomicrospira*, *Methylophaga*, *Pseudomonas*, *Pseudospirillum*, and *Pseudoalteromonas* belong to the class Gamma-proteobacteria and have a wide global distribution, suggesting that they have great adaptability to different environments [91,92].

### 4.3. Role of pH

The impact of pH on modulating the predominant phyla has been described [93]. A higher pH favors phyla such as Actinobacteria, Bacteroides, and Alpha/Beta/Gamma-proteobacteria. According to Ratzke [94], microorganisms can modify their environments, establishing interactions within single- and multi-species populations. Likewise, in silico studies have shown that pH indirectly affects the metabolism of microbial communities in anoxic environments by modulating the thermodynamics and kinetics of redox reactions [95,96]. The pH describes the chemical activity of protons, which are key in redox reactions, mineral precipitation and dissolution, and other geochemical reactions [97]. These reactions that are indirectly modulated by pH determine the salinity and composition of aqueous solutions and control the bioavailability of nutrients [98].

In our study, the most influential environmental factor that affected the diversity of bacterial communities within the La Brava–La Punta lake system was the pH of the water column. The pH pattern allowed us to differentiate samples from La Brava (mostly pH < 8.00) and La Punta (all pH ≥ 8.00; Table 2; Figure 2; Figure S1). As mentioned, B09 and B08 from La Brava exhibited similar conditions to those of the La Punta sampling points; these conditions led to a bacterial community composition with an increased abundance of the genus *Rubritalea* of phylum Verrucomicrobia. This observation was reinforced by PCoA and CAA, which separated the lakes into two well-defined groups (Table 2; Figure 2, Figure 3 and Figure 5; Figure S1). Our results are in agreement with previous studies on different lake systems that established the relevance of pH and how it can impact and define the composition of microbial communities in either the water column [64,93] or sediment [64,98].

The balance between the concentration of carbonates and bicarbonates modulates water pH levels in lakes [99]. The more alkaline a lake is, the greater the concentration of carbonates; however, the magnesium concentrations are reduced [100]. Low concentrations of magnesium and high concentrations of carbonates (Table 2) were found in the sampling points with the highest pH values (8.19–8.35), which can possibly explain the influence these parameters had on the genera detected in the main body of La Punta (P01–P04), such as *Pseudospirillum*, *Roseovarius*, *Pseudoalteromonas*, *Candidatus_Aquiluna*, *Balneola*, *Methylophaga*, *Alcanivorax*, *Vibrio*, *Perlucidibaca*, *Loktanella*, *Fabibacter*, and *Haliea*.

Even though B01 of La Brava and P05 and P06 of La Punta all have an alkaline pH (8.06, 8.07, and 8.17, respectively), the pH did not appear to influence the relative abundance of the genera found in these sampling points, as they all exhibited low carbonate concentrations. For this reason, DO may be more influential in shaping the relative abundance of the genera at B01 while silica concentrations influence the genera found at P05 and P06.

Although metabolic activity was not measured, it can be inferred that pH variations—the decrease or increase in environmental pH by one unit—can eventually decrease the metabolic activity of the microbial communities present by up to 50% in the La Brava–La Punta lake system [94].

## 5. Conclusions

This study is the first to simultaneously analyze the aquatic microbial diversity and composition of the La Brava and La Punta HAALs located in the southern section of the Salar of Atacama. We found that the phyla Proteobacteria, Bacteroidetes, and Actinobacteria accounted for more than 96% of the total relative abundance in the lake system. The α-diversity values, measured by the Shannon index, were higher for La Punta (4.11 ± 0.11) than La Brava (3.91 ± 0.26). The β-diversity index indicated that the sampling points for both HAALs were well distributed and that both lakes can be separated using the PCoA first component; however, B08 and B09 had a microbial composition similar to the La Punta HAAL.

The pH and EC, along with other physicochemical conditions, depends on the hydrological characteristics of the Monturaqui–Negrillar–Tilopozo aquifer as well as the underground water, which contains different ions and minerals. Analysis of the physicochemical parameters demonstrated that La Punta lake has a homogeneous pattern, unlike La Brava. In La Brava, most of the sampling points in the main body (B02–B07) shared similar parameters of pH < 7.95, EC > 40.00 mS/cm, DO < 2.00 mg/L, calcium > 400.00 mg/L, and magnesium > 1300 mg/L; however, they were dissimilar to sampling points from the isolated bodies (B08–B10) whose characteristics were instead similar to those of the main body of La Punta (P01–P04). Moreover, statistical analysis showed that only the pH and total silica were significantly different in both lakes. When comparing the main and isolated bodies of each lake, a significant difference was only observed in La Brava in terms of DO. Meanwhile, only between the main bodies were significant differences observed in pH, EC, salinity, DO, total silica, dissolved magnesium, the alkalinity of carbonates, and hardness.

Nevertheless, environmental parameters such as pH, EC, salinity, and carbonate alkalinity levels were important in conditioning the diversity of the lake system. When analyzing the entire lakes separately, the diversity of La Brava was not correlated with salinity concentration—despite the inlet of brackish water—but with pH alone. But, the division between the main body and isolated points did not present significant correlations.

According to our CCA, the complex ecological dynamics in the La Brava–La Punta lake system, which influenced the composition and distribution of the genera observed, were not only determined by pH and salinity but also by DO, silica, and dissolved magnesium and calcium^+^. These physicochemical parameters of the water column directly influenced the relative abundance of *Pseudospirillum*, *Roseovarius*, *Pseudoalteromonas*, *Candidatus_Aquiluna*, *Balneola*, *Methylophaga*, *Alcanivorax*, *Vibrio*, *Perlucidibaca*, *Loktanella*, *Fabibacter*, and *Haliea* in the La Punta sampling points.

In the future, an in-depth analysis of the microbial diversity and taxonomical composition of La Brava and La Punta lakes, while also considering spatio-temporal parameters, is required. Nevertheless, our study findings demonstrate how the microbial diversity in this ecological system is associated with the physicochemical characteristics of the water column. These findings can have potential implications for the biotechnology field down the line, including possible industrial applications that aim to solve economic-productive challenges such as the provision of fuels, mineral resources, or other products derived from bioprocesses; pharmaceuticals for obtaining new products or inputs of interest in the field of medicine; and environmental applications such as the use of biocatalysts or bioremediators for the recovery of contaminated areas or biofacilitators for reforestation and the recovery of degraded environments.

## Figures and Tables

**Table 1:** Characteristics of the hydrological and ecological dynamics of the La Brava-La Punta lake system and establishment of sampling points.

Lake	Sampling Points Characteristics		
	Hydrological Dynamics	Ecological Disaggregation Characteristics *	
	Inlet	Main Body	Isolated Body
La Brava	B01	B01 to B07	B08 to B10
La Punta	-	P01 to P04	P05 to P06

(*) Depending on water dynamics (inlet and output), disaggregation of the water bodies was classified as principals or isolated bodies. (-) denotes de absence of a water inlet into the lake.

**Table 2:** Physicochemical parameters of the water columns sampled from the La Brava–La Punta lake system in summer (December 2017).

Lake	Sampling Points	pH	DISS Oxygen (mg/L)	Salinity (g/L)	EC (mS/cm)	Total Silica (mg/L)	Ammonium (mg/L)	Nitrite (mg/L)	Nitrate (mg/L)	Total Nitrogen (mg/L)	P-PO4 Phosphates (mg/L)	Phosphorus (mg/L)	DISS Calcium (mg/L)	DISS Magnesium (mg/L)	Hardness (CaCO_3_ mg/L)	Alkalinity Carbonates mg/L CaCO_3_	Alkalinity Bicarbonates mg/L CaCO_3_^-^	Total Alkalinity mg/L CaCO_3_	TOC (mg/L)	Total Solids (mg/L)
La Brava	B01	8.06	3.09	19.08	30.32	68.90	<0.09	<0.01	0.25	1.00	<0.09	<0.05	219.88	633.20	3156.60	<1.00	317.40	317.40	5.00	5.00
	B02	7.64	1.73	52.84	77.32	91.80	<0.09	<0.01	1.03	1.70	<0.09	<0.05	805.00	2151.90	10,871.60	<1.00	556.90	556.90	15.20	4.00
	B03	7.55	1.61	50.87	74.67	74.60	<0.09	<0.01	0.63	31.20	0.11	0.13	441.80	1608.90	7728.60	<1.00	503.40	503.40	7.66	4.00
	B04	7.63	1.74	52.90	77.40	67.40	<0.09	<0.01	0.39	1.70	<0.09	<0.05	336.28	1340.10	6358.20	<1.00	409.00	409.00	6.25	6.00
	B05	7.62	3.94	77.84	110.40	86.60	<0.09	<0.01	1.05	3.10	<0.09	0.20	816.90	2347.50	11,706.80	<1.00	674.70	674.70	17.00	6.00
	B06	7.86	1.58	77.30	109.70	87.80	<0.09	<0.01	1.05	3.40	0.10	0.11	835.40	2,456.60	12,202.30	<1.00	674.70	674.70	17.40	4.00
	B07	7.92	3.00	27.70	42.70	89.60	<0.09	<0.01	0.96	2.40	<0.09	0.12	758.60	4545.00	20,610.50	<1.00	621.20	621.20	15.40	6.00
	B08	7.99	5.96	14.65	23.77	83.80	<0.09	<0.01	0.24	1.00	<0.09	<0.05	255.77	549.00	2899.40	46.90	381.30	428.20	5.53	5.00
	B09	8.18	6.35	22.39	35.11	86.30	<0.09	<0.01	0.38	0.50	<0.09	0.10	323.86	694.30	3667.80	89.50	379.10	468.60	7.14	5.00
	B10	7.64	4.82	50.87	47.28	78.00	<0.09	<0.01	0.44	2.10	<0.09	0.07	383.70	1070.50	5366.40	<1.00	385.60	385.60	5.00	4.00
La Punta	P01	8.19	4.01	23.40	36.57	127.40	<0.09	<0.01	0.50	1.80	<0.09	0.16	336.15	724.90	3824.50	10.70	334.40	345.10	7.64	4.00
	P02	8.21	3.71	23.46	36.66	127.60	<0.09	<0.01	0.53	1.40	<0.09	<0.05	358.44	729.70	3899.90	49.00	300.30	349.30	9.04	10.00
	P03	8.35	4.06	17.10	27.41	110.80	<0.09	<0.01	0.33	1.50	<0.09	<0.05	284.66	462.60	2615.80	63.90	305.70	369.60	5.84	3.00
	P04	8.27	4.25	13.08	21.42	100.50	<0.09	<0.01	0.24	1.10	<0.09	0.11	242.26	397.08	2240.10	36.20	317.40	353.60	14.50	5.00
	P05	8.07	3.14	40.10	60.00	103.80	<0.09	<0.01	0.85	2.80	<0.09	<0.05	340.20	771.35	4025.90	<1.00	488.80	488.80	11.70	6.00
	P06	8.17	3.55	40.93	61.15	107.20	<0.09	<0.01	0.95	3.80	<0.09	<0.05	484.50	1141.20	5909.30	<1.00	519.70	519.70	15.40	7.00
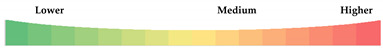																				

DISS: Dissolved ions, EC: electrical conductivity, TOC: total organic carbon. Color scales were applied per column (i.e., to each parameter) and from each lake, according to the lowest (green) and the highest (red) value. Columns with no variation were kept in green.

**Table 3:** Alpha diversity of the water column samples based on amplicon sequencing of the 16S rRNA gene hypervariable V3 and V4 regions (at ≥ 97% similarity).

Lake	Sample Points	Observed Out	Chao Index	Shannon Index	Simpson Index
La Brava	B01	1183	1234.43	4.24	0.95
	B02	1006	1085.33	3.98	0.92
	B03	935	1046.12	3.89	0.92
	B04	924	1093.36	3.62	0.87
	B05	985	1120.05	3.76	0.91
	B06	983	1104.11	3.88	0.93
	B07	896	936.67	3.90	0.93
	B08	948	1043.00	4.15	0.96
	B09	909	970.09	4.20	0.95
	B10	1186	1235.10	3.43	0.87
La Punta	P01	828	918.29	4.26	0.96
	P02	940	1071.26	4.04	0.95
	P03	799	901.69	4.22	0.96
	P04	835	954.02	4.09	0.95
	P05	887	997.53	3.98	0.94
	P06	868	992.32	4.07	0.95

**Table 4:** SIMPER analysis of the main operational taxonomic units (OTUs) associated with the differences between La Brava and La Punta lakes.

La Brava-La Punta		La Brava		La Punta		Main		Isolated	
		(Main-Isolated)		(Main-Isolated)		La Brava-La Punta		La Brava-La Punta	
Genus	%Cum Sum	Genus	%Cum Sum	Genus	%Cum Sum	Genus	%Cum Sum	Genus	%Cum Sum
*Psychroflexus*	26.37	*Psychroflexus*	18.48	*Thiomicrospira*	20.51	*Psychroflexus*	23.65	*Psychroflexus*	23.87
*Thiomicrospira*	36.34	*Pseudospirillum*	28.07	*Psychroflexus*	40.47	*Thiomicrospira*	44.82	*Pseudomonas*	37.46
*Pseudospirillum*	45.83	*Rubritalea*	37.55	*Pseudomonas*	56.69	*Pseudospirillum*	52.17	*Pseudospirillum*	45.93
*Pseudomonas*	52.77	*Pseudomonas*	45.51	*NS5 marine group*	60.92	*Roseovarius*	56.67	*Rubritalea*	53.63
*Roseovarius*	58.15	*Pseudoalteromonas*	52.08	*Pseudoalteromonas*	64.51	*Pseudomonas*	60.88	*Pseudoalteromonas*	58.76
*Ruegeria*	61.85	*Roseovarius*	57.26	*Ruegeria*	68.01	*Pseudoalteromonas*	64.91	*NS5 marine group*	62.27
*Pseudoalteromonas*	65.42	*Kiloniella*	60.55	*Pseudospirillum*	71.17	*Ruegeria*	68.62	*Thiomicrospira*	65.42
*Rubritalea*	68.74	*Candidatus_Aquiluna*	63.81	*DS001*	73.16	*DS001*	71.00	*Ruegeria*	68.34
*Candidatus_Aquiluna*	71.28	*Thiomicrospira*	66.80	*Roseibaca*	75.13	*Roseibaca*	73.21	*Roseovarius*	71.05
*Roseibaca*	73.41	*Ruegeria*	69.58			*Marinicella*	75.05	*Roseibaca*	73.61
*DS001*	75.36	*DS001*	71.70					*Kiloniella*	76.01
		*NS5 marine group*	73.48						
		*Perlucidibaca*	75.09						

% Cum Sum: % cumulative sum.

## Supplementary Materials

The following are available online at https://www.mdpi.com/2076-2607/8/8/1181/s1, Figure S1: The α-diversity of microorganisms present in the water columns of the La Brava and La Punta lake system, Figure S2: Cluster analysis of the different sampling points of the La Brava–La Punta lake system using the Ward method, based on dissimilarities of β-diversity, Table S1: Comparison between lakes and physicochemical parameters of the water column samples obtained from the La Brava–La Punta lake system during the summer (December 2017), Table S2: Raw data summary generated by MiSeq using demultiplexing data for sampling, Table S3: Influence of environmental physicochemical parameters on the α-diversity of the La Brava-La Punta lake, Table S4: Relative abundance of the first 31 genera in the La Brava and La Punta lake system, Data S1: Genera in La Brava and La Punta lakes at Salar de Atacama, Chile.
