# Supplementary material for: Physicochemical Parameters Affecting the Distribution and Diversity of the Water Column Microbial Community in the High-Altitude Andean Lake System of La Brava and La Punta
Source: Microorganisms. 2020 Aug 3;8(8):1181. doi: 10.3390/microorganisms8081181 (PMC7464526; doi:10.3390/microorganisms8081181)
Supplement: Supplementary file 1 [file microorganisms-08-01181-s001.pdf]

Supplemental Data

Genera in La Brava and La Punta lakes

Phylum: Proteobacteria - Class: Gammaproteobacteria

Methylophaga comprise halophilic methylotrophic bacteria, which are normally isolated from marine environments or environments with low water activity, such as hypersaline lakes. These bacteria play a key role in the biogeochemical cycles of methanol, mono-methylamine, dimethylsulfide, and methyl bromide.

Pseudoalteromonas are gram-negative bacteria that have flagella. This genus contains numerous marine species, which synthesize biologically active molecules. These bacteria are usually found in association with marine eukaryotes, and have antibacterial, bacteriolytic, agrolytic, and algacidal activity. Their antibacterial activity allows them to colonize surfaces. The production of bacteriolytic substances, such as agaroses and toxins, allows them to compete for nutrients and space, as well as to protect themselves from possible predators. The bacteria have a great competitive advantage in the acquisition of nutrients and colonization, due to their lethal activity against phytoplankton. This provides ecological relief, since it controls the succession of phytoplankton in marine environments. The bacteria live in a wide variety of habitats globally, suggesting great adaptability and successful survival strategies.

Pseudomonas are gram negative bacteria that are motile with a flagellum. There are approximately 200 species. Their metabolic diversity allows them to colonize a wide range of niches, For example: *Pseudomonas aeruginosa* is an opportunistic pathogen, *P. syringae* is a plant pathogen, and *P. putida* is an efficient inhabitant of soils. Many members of this genus can adapt to natural environments, such as soil.

Pseudospirillum is a gram-negative bacteria genus. The bacteria are mobile by means of a flagellum, form spores, are halophilic, aerobic, chemoheterotrophic, oxidase positive, and catalase negative.

Thiomicrospira are moderately psychrophilic, aerobic, autologous chemolytic, and can oxidize sulfur and hydrogen. This latter activity is related to the concentration of nickel in the medium. This bacterium has been detected in several environments, including hydrothermal vents at the sea floor. This bacterial genus fixes carbon in hydrothermal vents, coastal sediments, hypersaline lakes and other sulfidic environments, and has a surprising diversity of mechanisms for dissolving inorganic carbon.

Phylum: Proteobacteria. Class: Alphaproteobacteria

Roseovarius. This genus is a member of the *Rhodobacteraceae* family. By 2014, 18 species had been recognized. Members of this genus have been isolated from several marine habitats, such as seawater hypersaline, marine sediments, and even algal cells. Tolerance and adaptation to extreme environments make them potential candidates for biotechnological purposes.

Ruegeria is genus of bacteria that are gram negative, aerobic, with oxidase and catalase activity. They are not photosynthetic and require NaCl for growth. The genus is composed of several species, which includes *R. atlantica*, *R. lacuscaerulensis*, and *R. pomeroyi*. These three species have been isolated from salty environments. *R. mobilis* has been isolated from the surface of the ocean water of the most diverse climatic zones, except in the waters of the Arctic and Antarctic.

Phylum: Bacteroidetes. Class: Flavobacteria

Psychroflexus belongs to the *Flavobacteriaceae* family. It is a group of psychrophilic bacteria that synthesize polyunsaturated fatty acids, such as eicosapentaenoic acid (20: 5, Omega 3) and arachidonic acid (20: 4, Omega 6). They have been found in closely related groups to moderate halophiles, with 98% sequence similarity. They also display 95.4% sequence similarity with *Phychroflexus halocasei*. In 2014, Chun described *Psychroflex salarius* sp., a bacterium that was isolated

at the Gomo salina in Korea. The bacterium is gram negative, moderately halophilic, with an optimal pH and temperatures of 25 and 6.5°C, respectively. It also has catalase and oxidase activity, and produces carotenoid pigment.

Balneola is a genus of bacteria that are aerobic, gram negative, and capable of living in a wide temperature range (10-40°C). The optimum pH is 8.0 and the salinity range is 0-50 g/L. They are capable of growing and living in extreme environments. These bacteria have been isolated from surface waters of the Mediterranean Sea. They require oxygen to oxidize substrate and generate energy. They are able to use N-acetylgalactosamine, adonitol, arabitol, arabinose, fructose, fucose, lactulose, maltose, and sorbitol as energy sources.

Phylum: Actinobacteria - Class: Actinobacteria

Candidatus_Aquiluna. It was once believed that this only grew in fresh water. However, it has also been isolated from saline environments. It is part of the *Microbacteriaceae* family.

DS001 is a genus of the *Microbacteriaceae* family. It has been isolated from marine environments and also from hypersaline lakes located in deserts.

Phylum: Verrucomicrobia. Class: Verrucomicrobiae

Rubritalea is an obligate marine bacterium that is gram negative, round, and non-mobile. It has an aerobic and chemoorganotrophic metabolism. *R. marina* is able to grow using glucose, xylose, melibiose, or cellobiose as the sole source of energy in aerobic conditions. It cannot grow anaerobically.

Table S1. Comparison between lakes and physicochemical parameters of the water column samples obtained from the La Brava–La Punta lake system during the summer (December 2017).

Variable	La Brava - La Punta		La Brava (main - isolated)		La Punta (main - isolated)		Main La Brava - La Punta		Isolated La Brava - La Punta	
	H	p-value	H	p-value	H	p-value	H	p-value	H	p-value
pH	9.22	0.0009*	2.55	0.1417	3.43	0.1333	7.00	0.0061*	0.33	0.8000
Dissolved Oxygen (mg/L)	0.95	0.3676	5.73	0.0167*	3.43	0.1333	6.04	0.0121*	3.00	0.2000
Salinity (g/L)	2.65	0.1179	2.55	0.1333	3.43	0.1333	5.14	0.0242*	0.33	0.8000
EC (mS/cm)	1.99	0.1806	2.92	0.1167	3.43	0.1333	5.14	0.0242*	3.00	0.8000
Total Silica (mg/L)	10.59	0.0002*	0.12	0.8333	0.86	0.5333	7.00	0.0061*	3.00	0.2000
Ammonium (mg/L)	-	-	-	-	-	-	-	-	-	-
Nitrite (mg/L)	-	-	-	-	-	-	-	-	-	-
Nitrate (mg/L)	0.36	0.5794	2.92	0.1083	3.43	0.1333	2.89	0.1030	3.00	0.2000
Total Nitrogen (mg/L)	0.00	> 0.9990	0.00	> 0.9999	3.43	0.1333	2.29	0.1364	3.00	0.2000
P-PO_4 Phosphates (mg/L)	0.42	0.5000	0.47	0.5333	-	-	0.57	0.4909	-	-
Phosphorus (mg/L)	0.50	0.4895	0.47	0.5500	0.86	0.4667	0.08	0.7879	1.33	0.4000
DISS Calcium (mg/L)	0.20	0.6354	2.19	0.1833	1.93	0.5333	2.89	0.1091	1.33	0.4000
DISS Magnesium (mg/L)	3.40	0.0643	3.75	0.0667	3.43	0.1333	5.14	0.0242*	1.33	0.4000
Hardness (mg/L)	3.40	0.0643	3.75	0.0667	3.43	0.1333	5.14	0.0242*	1.33	0.4000
Alkalinity carbonates mg/L $CaCO_3$	1.69	0.1384	2.55	0.0667	3.43	0.1333	7.00	0.0030*	1.33	0.4000
Alkalinity bicarbonates mg/L $CaCO_3$	3.60	0.5990	2.92	0.1083	3.43	0.1333	5.58	0.1520	3.00	0.2000
Total Alkalinity mg/L $CaCO_3$	2.65	0.1130	1.57	0.2500	3.43	0.1333	3.57	0.0697	3.00	0.2000
TOC (mg/L)	0.14	0.7319	2.55	0.1250	1.93	0.5333	0.89	0.4121	3.00	0.2000
Totals Solids (mg/L)	0.42	0.5260	0.21	0.6917	0.86	0.5333	0.14	0.6606	3.00	0.1000

La Brava: La Brava medians, La Punta: La Punta medians, H: H-statistic for Kruskal-Wallis, DISS: Dissolved, EC: electrical conductivity, TOC: total organic carbon. (*) Represents statistical significance (p-value < 0.05). (-) denotes comparisons not made to show no variation.

Table S2. Raw data summary generated by MiSeq using demultiplexing data for sampling.

Lake	Sampling points	Length	Number of Raw Reads	Numbers of Bases	Q20(%)	Q30(%)	Pair-end after filtering	No Chimera after filtering	CG (%)
La Brava	B01	250	2,030,906	507,726,500	97.58	95.56	1,015,453	805,032	51.94
	B02	250	1,729,514	432,378,500	97.76	95.87	864,757	709,508	49.97
	B03	250	1,699,904	424,976,000	97.18	94.88	779,914	619,301	50.80
	B04	250	2,172,562	543,140,500	96.34	93.51	1,086,281	915,064	50.62
	B05	250	1,518,976	379,744,000	97.44	95.33	759,488	599,288	50.92
	B06	250	1,699,904	424,976,000	97.18	94.88	849,952	665,214	51.04
	B07	250	1,832,122	458,030,500	97.66	95.70	916,061	726,066	51.03
	B08	250	1,732,300	433,075,000	97.70	95.77	866,15	720,918	52.32
	B09	250	2,179,438	544,859,500	97.06	94.71	1,089,719	768,862	51.85
	B10	250	1,720,590	430,147,500	97.40	95.28	860,295	717,529	51.84
La Punta	P01	250	1,413,826	353,456,500	97.06	94.69	706,913	393,33	53.50
	P02	250	1,445,924	361,481,000	97.51	95.43	722,962	526,899	54.29
	P03	250	1,199,502	299,875,500	96.79	94.22	599,751	504,548	53.84
	P04	250	1,807,074	451,768,500	97.28	95.05	903,537	790,82	53.27
	P05	250	2,175,272	543,818,000	97.69	95.74	1,087,636	789,538	52.83
	P06	250	1,224,784	306,196,000	97.35	95.13	612,392	501,251	54.10

Table S3. Influence of environmental physicochemical parameters on the α-diversity of the La Brava-La Punta lake.

Factor	Shannon index													
	Lakes system		La Brava		La Brava Main Water		La Brava Isolated Water		La Punta		La Punta Main Water		La Punta Isolated Water	
	Pearson	p-value	Pearson	p-value	Pearson	p-value	Pearson	p-value	Pearson	p-value	Pearson	p-value	Pearson	p-value
pH	0.77	0.0004*	0.75	0.0116*	0.67	0.0975	0.96	0.1885	0.61	0.2022	0.16	0.8423	-	-
EC	−0.55	0.0288*	−0.44	0.1990	-0.61	0.1438	-0.85	0.3573	−0.53	0.2800	0.14	0.8663	-	-
Salinity	-0.68	0.0034*	-0.62	0.0558	-0.61	0.1498	-0.97	0.1670	-0.56	0.2756	0.13	0.8636	-	-
Ox diss	0.30	0.2534	0.25	0.4817	0.16	0.7323	0.98	0.1208	0.70	0.1212	0.32	0.6840	-	-
Ca diss	−0.37	0.1613	−0.23	0.5215	-0.24	0.6021	-0.81	0.3940	−0.27	0.6110	-0.01	0.9930	-	-
Mg diss	−0.39	0.1374	−0.22	0.5357	-0.22	0.6298	-0.95	0.2110	−0.32	0.5399	0.04	0.9588	-	-
Carbonate	0.51	0.0414*	0.54	0.1090	0.00	>0.9990	0.90	0.2827	0.30	0.5643	-0.33	0.6726	-	-
Bicarbonate	−0.41	0.1174	−0.23	0.5195	-0.38	0.4051	-0.96	0.1790	−0.54	0.2894	0.63	0.3733	-	-

(*) Represents statistical significance (p-value < 0.05). (-) denotes comparisons not made due to the lack of points.

The Pearson correlation and corresponding p-value between α-diversity (Shannon index) and the main physicochemical parameters are shown. EC: electrical conductivity; Ox diss: dissolved oxygen; Ca diss: dissolved calcium; Mg diss: dissolved magnesium; carbonate: alkalinity carbonate; bicarbonate: alkaline bicarbonate. Only 8 of the most constraining variables are shown in this table.

Table S4. Relative abundance of the first 31 genera in the La Brava and La Punta lake system.

Genera	Relative abundance (%)															
	La Brava										La Punta					
	B01	B02	B03	B04	B05	B06	B07	B08	B09	B10	P01	P02	P03	P04	P05	P06
Psychroflexus	14.63	51.05	60.65	8.10	64.90	57.04	54.39	0.83	0.31	56.61	0.10	0.19	0.08	0.02	0.49	1.04
Pseudospirillum	1.95	0.16	0.06	0.94	0.02	0.04	0.30	16.70	6.18	0.29	8.83	18.67	28.23	26.88	15.38	26.40
Roseovarius	3.94	2.84	4.15	2.24	3.53	3.54	3.47	6.11	6.01	2.94	21.92	36.34	17.86	2.65	8.56	15.87
Thiomicrospira	2.54	20.94	7.23	64.45	3.39	12.02	11.79	0.07	0.00	2.40	0.01	0.71	0.00	0.00	0.01	0.13
Pseudomonas	36.81	1.77	0.03	2.93	0.92	0.42	0.07	5.19	19.81	10.09	8.59	3.31	7.55	22.34	0.41	0.63
Pseudoalteromonas	0.54	0.06	0.01	0.27	0.09	0.06	0.01	13.03	11.87	0.43	4.95	0.25	1.65	0.24	19.19	7.44
Ruegeria	0.69	9.11	8.62	0.22	8.10	7.37	7.74	0.01	0.01	10.10	0.02	0.07	0.01	0.00	0.12	0.27
Rubritalea	0.02	0.01	0.00	0.33	0.03	0.00	0.00	12.69	20.80	0.11	0.01	0.02	4.83	5.42	0.36	4.95
DS001	0.04	2.02	5.65	0.01	6.42	6.07	6.89	0.10	0.12	0.01	0.48	0.50	0.48	0.45	4.68	7.20
Candidatus_Aquiluna	0.07	0.21	0.50	0.00	0.57	0.55	0.53	0.81	0.54	0.48	2.69	3.42	10.50	12.54	0.71	0.85
Roseibaca	1.80	4.74	6.22	0.72	5.30	4.59	5.88	0.01	0.07	0.50	0.02	0.01	0.01	0.00	0.27	0.53
NS5_marine_group	9.63	0.00	0.00	0.00	0.00	0.00	0.00	1.04	4.09	2.31	10.95	0.03	0.00	0.01	0.01	0.00
Balneola	0.01	0.00	0.00	0.01	0.00	0.00	0.00	0.40	0.52	0.01	2.66	4.32	3.21	1.89	3.19	4.14
Marinomonas	0.93	0.02	0.00	0.00	0.00	0.01	0.00	1.44	2.87	0.77	5.56	0.09	0.86	0.03	6.90	0.54
Methylophaga	0.76	0.00	0.00	0.32	0.01	0.00	0.00	2.29	2.37	0.12	3.20	9.05	0.64	0.12	0.66	0.08
Alcanivorax	0.02	0.01	0.00	0.41	0.02	0.01	0.03	3.44	2.61	1.08	2.51	0.01	0.01	0.07	3.86	4.68
Perlucidibaca	2.21	0.00	0.00	0.00	0.00	0.00	0.00	2.72	0.81	0.17	0.54	1.39	1.24	7.02	0.00	0.00
Owenweeksia	0.09	1.14	0.62	1.20	0.22	0.61	0.85	2.13	2.24	0.54	1.01	0.64	0.68	1.99	0.55	0.81
Marinicella	0.20	0.05	0.03	0.56	0.03	0.01	0.01	0.01	0.19	0.19	0.13	0.06	0.01	0.01	8.31	5.36
Loktanella	0.24	0.12	0.44	0.07	0.40	0.48	0.51	1.62	0.55	1.55	1.42	2.39	1.96	2.82	0.16	0.02
Marivita	0.07	0.34	1.81	0.15	1.85	2.11	2.43	0.03	0.11	0.05	0.05	0.55	0.14	0.11	3.27	1.27
Hyphomonas	3.85	0.20	0.06	0.07	0.07	0.04	0.05	2.97	1.48	0.84	1.04	1.11	0.88	1.26	0.15	0.22
Arthrobacter	0.30	0.49	0.96	0.00	1.15	1.11	0.86	0.53	0.23	1.80	1.09	1.37	0.52	0.30	1.38	1.39
Kiloniella	0.14	0.00	0.00	0.01	0.01	0.00	0.00	8.27	3.23	0.01	0.01	0.08	0.94	0.03	0.02	0.00
Fabibacter	0.04	0.02	0.01	0.55	0.01	0.02	0.05	1.16	2.10	0.25	1.05	0.67	1.29	0.68	2.93	1.32
Others	18.49	4.71	2.94	16.44	2.95	3.87	4.15	16.42	10.87	6.34	21.15	14.74	16.39	13.13	18.44	14.85

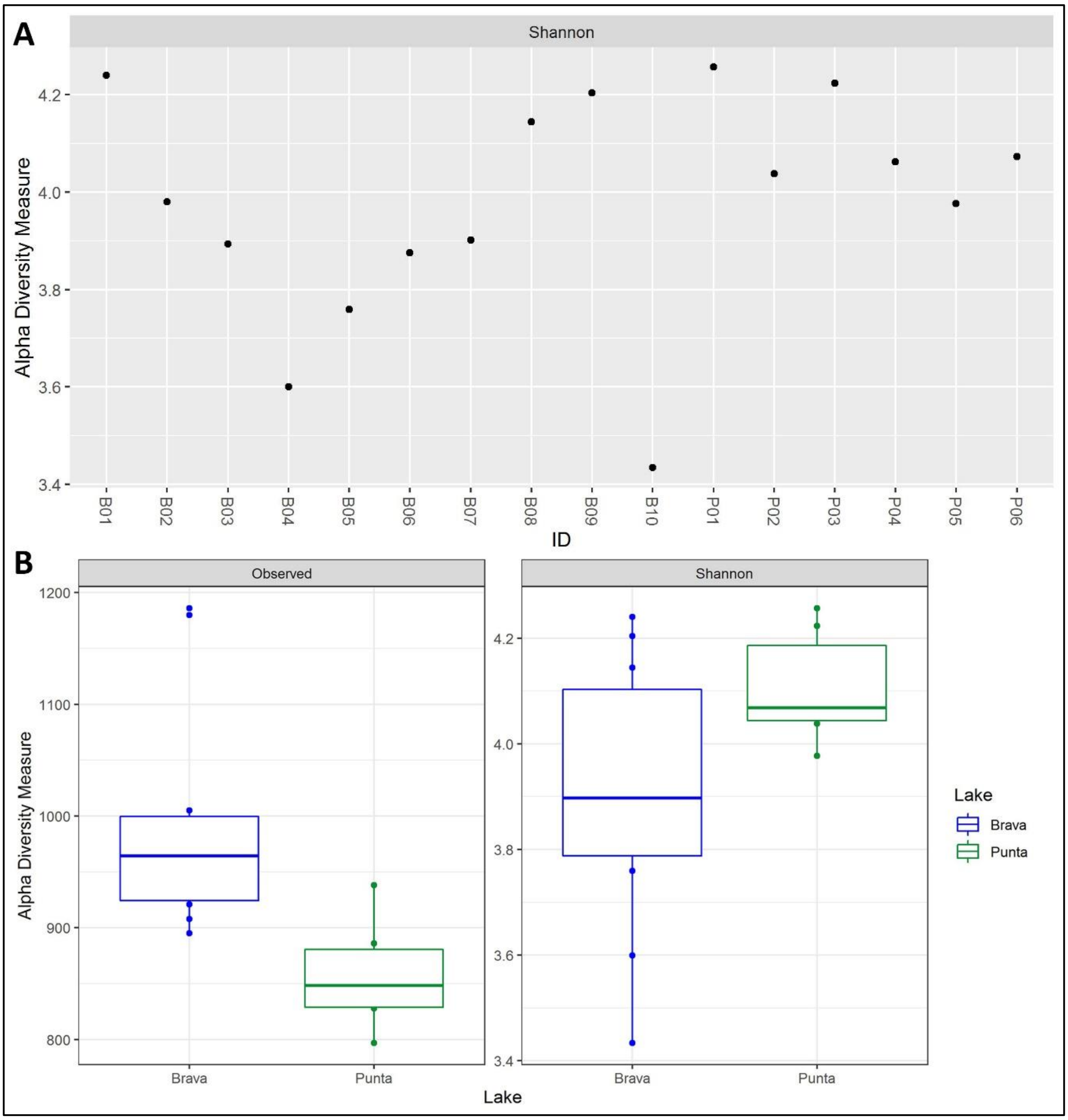

Figure S1. The α-diversity of microorganisms present in the water columns of the La Brava and La Punta lake system.

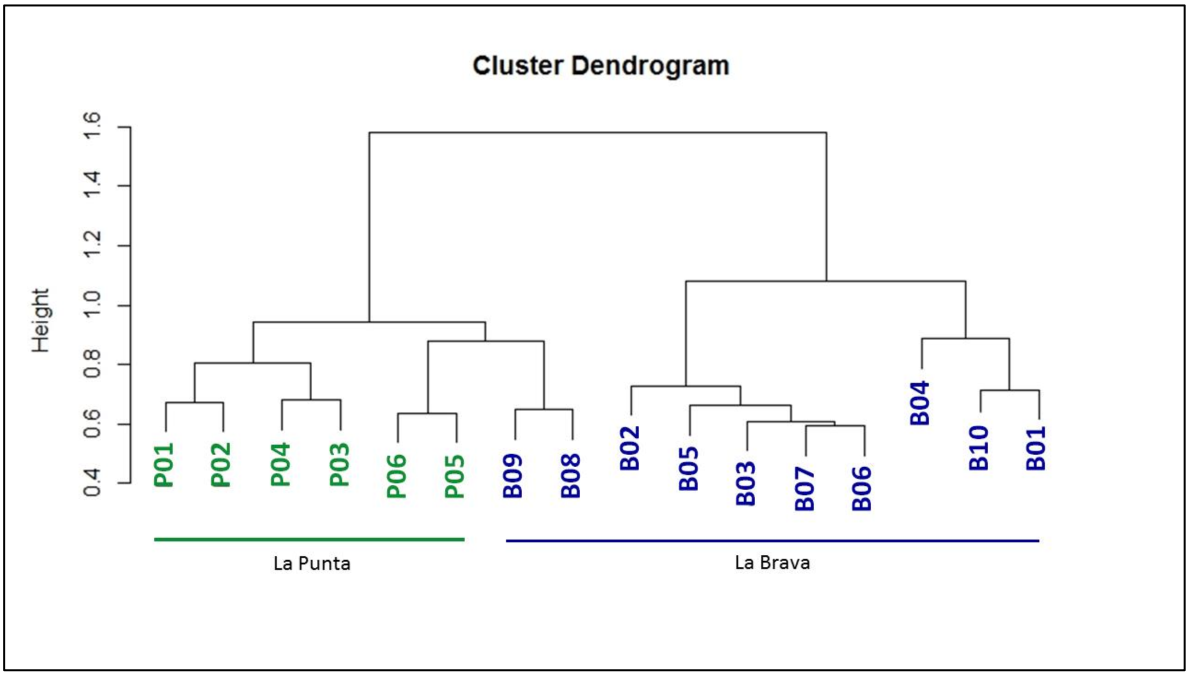

Figure S2. Cluster analysis of the different sampling points of the La Brava–La Punta lake system using the Ward method, based on dissimilarities of β-diversity.
